# Human Remains: Curation, Reburial and Repatriation

**DOI:** 10.5195/jmla.2024.1525

**Published:** 2024-01-16

**Authors:** Kevin O'Brien

**Affiliations:** 1 kevinm@uic.edu, University of Illinois at Chicago, Chicago, IL

Libraries share many institutional characteristics with museums. Library special collections and archives departments often contain items that would be on display or stored in a museum. Health sciences libraries with collections that include anatomical items may face similar challenges as museums that include human remains in their holdings, and documentation of provenance as well as the ethics of organizing exhibits. As the level of professional awareness of the ethical and cultural issues in the management of human remains increases, health sciences librarians and archivists may need resources to help make informed professional decisions.[1]

Margaret Clegg's, *Human Remains: curation, reburial and repatriation* is a comprehensive overview of the issues involved in the archival management of human anatomical material. Clegg, formerly of the Natural History Museum in London, is steeped in the complexities involved in this topic and shares her experience in a comprehensive examination of its many facets. While most of the examples are drawn from the institutional, legal, and historical context of the United Kingdom, the United States milieu is also included.

Clegg discusses the way museums have acquired human remains for their collections. In the past, human remains were often taken during military campaigns or exploratory endeavors without respect for the concept of consent. Subsequent decades of decolonization as well as cultural and intellectual change have altered the relationship between the remains, their institutional custodians, and the communities from which they were taken.

Clegg takes up in detail the legal framework governing human remains with a predominant focus on UK law. For comparative purposes, the details of the UK's 2004 Human Tissue Act will be of interest to those familiar only with US laws like the Native American Graves Protection and Repatriation Act of 1990.

The chapter on ethical issues provides important context to the management of research involving human remains on display. The author defines the concept of ethics, offers approaches to distinguishing qualitative categories of remains based on date of death, and discusses professionally accepted museum curatorial values that can serve as a guide to other professions in managing research and display of remains.

Drawn from experience with remains from Tasmania and the Torres Strait Islands, the author shares some of the most interesting material in the book; the issue of repatriation of remains to their original communities. Clegg openly describes the positives and negatives of the two cases, including a successful effort to develop relations with representatives of the Torres Strait Islanders to make the repatriation process an inclusive one.

Content review questions are provided at the end of each chapter, making the book appropriate for inclusion in relevant course curricula. While the variety and complexity of human remains faced by library special collections departments is far less likely than natural history museums, this book has relevance for the library world. The historical and professional context provided by Clegg will benefit librarians and archivists and will support anthropology, biomedical ethics, and forensics curricula.

## References

[1] Gazi, A 2014 Exhibition Ethics - An Overview of Major Issues. Journal of Conservation and Museum Studies, 12(1): 4, pp. 1-10, DOI: 10.5334/jcms.102121

